# Differential Recruitment of the Infralimbic Cortex in Recent and Remote Retrieval and Extinction of Aversive Memory in Post-Weanling Rats

**DOI:** 10.1093/ijnp/pyac012

**Published:** 2022-02-04

**Authors:** Walaa Awad, Milly Kritman, Guillaume Ferreira, Mouna Maroun

**Affiliations:** Department of Neurobiology, Faculty of Natural Sciences, University of Haifa, Haifa, Israel; Department of Neurobiology, Faculty of Natural Sciences, University of Haifa, Haifa, Israel; Univ. Bordeaux, INRAE, Bordeaux INP, NutriNeuro, UMR 1286, Bordeaux, France; Department of Neurobiology, Faculty of Natural Sciences, University of Haifa, Haifa, Israel

**Keywords:** IL-mPFC, remote memory, conditioned odor aversion, contextual fear conditioning, juvenility

## Abstract

**Background:**

We previously showed that the infralimbic medial prefrontal cortex (IL-mPFC) plays an important role in recent and remote memory retrieval and extinction of conditioned odor aversion (COA) and contextual fear conditioning (CFC) in adult rats. Because the mPFC undergoes maturation during post-weaning, here, we aimed to explore (1) whether post-weanling rats can form recent and remote COA and CFC memory, and (2) the role of the IL-mPFC in mediating these processes.

**Methods:**

To investigate the retrieval process, we transiently inactivated the IL-mPFC with lidocaine prior to the retrieval test at either recent or remote time points. To target the consolidation process, we applied the protein synthesis inhibitor after the retrieval at recent or remote time points.

**Results:**

Our results show that the post-weanling animals were able to develop both recent and remote memory of both COA and CFC. IL-mPFC manipulations had no effect on retrieval or extinction of recent and remote COA memory, suggesting that the IL has no effect in COA at this developmental stage. In contrast, the IL-mPFC played a role in (1) the extinction of recent, but not remote, CFC memory, and (2) the retrieval of remote, but not recent, CFC memory. Moreover, remote, but not recent, CFC retrieval enhanced c-Fos protein expression in the IL-mPFC.

**Conclusions:**

Altogether, these results point to a differential role of the IL-mPFC in recent and remote CFC memory retrieval and extinction and further confirm the differences in the role of IL-mPFC in these processes in post-weanling and adult animals.

**Significance Statement**

The medial prefrontal cortex is one of the brain structures important for higher cognitive functions and emotional regulation. The medial prefrontal cortex is characterized by ongoing maturation even after puberty in humans and after weaning in rodents. This protracted maturation may suggest that its function in regulating cognitive and emotional processes may not be similar in the adult animal and the juvenile animal. This study aimed to address the role of the medial prefrontal cortex in 2 types of memories that were previously reported to require the medial prefrontal cortex: consolidation of extinction memories and storage of remote memories that, after their formation, are transferred to the cortex.

Using inactivation as well as blockade of protein synthesis to gain insight into its role during different memory processes, we report that different types of memory processes are processed differently in adults and juvenile animals.

## Introduction

It has been suggested that infantile amnesia is due to the underdevelopment of the infant brain, which would preclude memory consolidation or memory retrieval (Alberini and Travaglia, 2017). In rodents, the adult-like fear response emerges during the second week after birth together with the maturation of the amygdala, a region critical for threat response processing (Bouwmeester et al., 2002a; Shionoya et al., 2006; Raineki et al., 2012), suggesting that the mechanisms underlying fear in young and adult animals may be similar at a very early stage. However, researchers previously showed that while young rodents can learn fearful events, they rapidly forget memories (Campbell and Campbell, 1962; Siette et al., 2014; Robinson-Drummer and Stanton, 2015; Ramsaran et al., 2016, 2019; Guskjolen et al., 2017). These patterns of amnesia in young animals are completely dissociated from the ability of adult animals to retain and recall memories, especially those that are aversive.

The interaction between the amygdala and the infralimbic part of the medial prefrontal cortex (IL-mPFC) was reported to be critical for the inhibition or extinction of recently acquired fear responses in adult rats (for reviews, see Sotres-Bayon and Quirk, 2010; Herry and Johnasen, 2014). Moreover, we and others reported that while IL-mPFC is involved in extinction of both recent (1–3 days after acquisition) and remote (1 month after acquisition) fear-based contextual fear conditioning (CFC), the IL-mPFC plays a distinct role in recent and remote CFC retrieval (Restivo et al., 2009; Vetere et al., 2011; Kritman and Maroun, 2013; Rosas-Vidal et al., 2014; Awad et al., 2015).

We previously extended the investigation of the role of IL-mPFC to extinction of non–fear-based aversive memories such as conditioned odor aversion (COA) and taste aversion as inhibition of protein synthesis in the IL-mPFC impaired extinction of recent aversion memory (Akirav et al., 2006; Awad et al., 2015). Learned flavor aversion is considered hippocampus independent (Josselyn et al., 2004; Cui et al., 2005; Ding et al., 2008; Ballarini et al., 2009) in contrast to CFC, which is hippocampus dependent (Goshen et al., 2011; Zelikowsky et al., 2012). Interestingly, we showed in adult rats that blocking protein synthesis in the IL-mPFC impaired extinction consolidation of recent, but not remote, COA, whereas it induced extinction deficits of both recent and remote CFC (Awad et al., 2015). Altogether, these results revealed in adults the essential but complex role of the IL-mPFC in retrieval and extinction of recent and remote aversive memory.

When comparing the role of the IL-mPFC in adult and young animals in mediating fear extinction, research focused on fear-based associations. It was reported that whereas the inactivation of the IL-mPFC did not affect the recall of fear extinction in pre-weanling rats, its inactivation resulted in impaired fear extinction in post-weanling and adult animals (for reviews, see Pattwell et al., 2013; Zimmermann et al., 2019). These results put forward the idea that the engagement of the IL-mPFC in the adult and the post-weaning animals is similar. Yet, the IL-mPFC still undergoes maturation during the post-weaning period, and we previously reported that some mechanisms are not identical in the 2 groups of age (Schayek and Maroun, 2015; Slouzkey and Maroun, 2016). According to the fact data are scarce about the role of the IL-mPFC in recent and remote extinction memory in juveniles, this study sought to address whether juvenile animals can form remote memories of either COA or CFC and to examine the role of the IL-mPFC in recent and remote retrieval and extinction of both COA and CFC learned in the post-weaning period (postnatal day [PND] 25–30). For this purpose, we used a combination of behavior, local pharmacological manipulations (transient inactivation or inhibition of protein synthesis), and neuronal activity markers (c-Fos western blot). Our findings point to a clear dissociation between COA and CFC in the role played by the IL-mPFC. They also hint at different time scales for maturation of the IL-mPFC circuits during adolescence that underlie retrieval and extinction of CFC memory.

## MATERIALS AND METHODS

### Animals

The experiments were conducted on male Sprague Dawley post-weaning rats aged PND 21–27 weighing 70–100 g. All subjects were weaned at PND 20 and caged 4–6 per cage, except during COA experiments, where they were housed individually at 22 ± 2°C under 12-hour-light/-dark cycles (lights on at 7:00 am). All tests were conducted between 9:00 am and 3:00 pm and were approved by the University of Haifa Ethics and Animal Care Committee. Adequate measures were taken to minimize pain and discomfort in accordance with US NIH guidelines. All rats underwent a handling familiarization session before the experiment began.

### Surgery

For the experiments examining recent memory, surgery of implantation of cannulae was performed at PND 22–24. For the remote testing, implantation of cannulae was performed at PND 53–55 to prevent clogging for cannulae, as we have previously reported (Awad et al., 2015). The young animals were given 3 days to recover before being subjected to experimental manipulations. This period was found to be sufficient for complete recovery in juvenile rats (Kim and Richardson, 2008; Slouzkey and Maroun, 2016). When surgery was conducted at PND 53–55, animals were given 7 days for recovery (Awad et al., 2015).

Rats were anesthetized via i.p. injection of ketamine (100 mg/kg) and xylazine (7.5 mg/kg). They were restrained in a stereotaxic apparatus (Stolting, Wood Dale, Illinois USA) and implanted bilaterally with stainless-steel guide cannulae (23-gauge, outer diameter 0.025/inner diameter 0.017) aimed at the IL-mPFC (anteroposterior: +2.7 mm from bregma; lateral: ±0.5 mm from bregma; ventral: −3.8 mm from bregma) in accordance with the rat brain atlas (Paxinos and Watson, 1998). The cannulae were held in place with acrylic dental cement and secured with 2 skull screws. A stylus was placed in each of the guide cannulae to prevent clogging. After the surgery, the rats received an antibiotic (0.05 mL Pen-strep 20/25 veterinary) and a painkiller (Calmagin at 0.2 mL/100 g).

### Behavior

The behavioral paradigms were conducted according to well-established protocols as described in our previous reports (Awad et al., 2015; Kritman and Maroun, 2017). Conditioning in each paradigm was conducted on PND 27, as described below.

#### COA Acquisition and Extinction

**—**On the first day of the experiment (Day 1: PND 24), rats housed individually were deprived of water for 24 hours. Water deprivation starting during recovery from surgery was found not to impede recovery or affect later performance in the behavioral tasks (Slouzkey et al., 2013; Awad et al., 2015).

Over the next 3 consecutive days (training: Day 2–3: PND 25–26), they were provided access to 2 pipettes, each containing 10 mL of tap water, for 20 minutes each day in their home cage. On the conditioning day (Day 4: PND 27), the water was replaced with two 10-mL pipettes of banana solution (0.1% w/v), and rats were given access to it for 20 minutes as previously. Forty minutes later, the rats were given i.p. injections of lithium chloride (0.15 M, 2% body weight) to induce malaise.

The banana-scented water was used in previous studies (Slotnick et al., 1997; Chapuis et al., 2007; Desgranges et al., 2009; Sevelinges et al., 2009a, 2009b) and did not induce any neophobic responses at this concentration during the first presentation. Moreover, it has been established that anosmic rats, lacking a sense of smell, are unable to detect 0.1% banana-scented water, whereas the same rats preformed normally in taste detection tests (Slotnick et al., 1997). Importantly, inactivating the gustatory cortex leads to impairment of taste aversion memory but not of COA using banana-scented water. Altogether, these data indicate that the processing of this banana-scented solution was mediated by odor rather than taste properties (Slotnick et al., 1997).

For recent memory COA tests, on the 2 days following conditioning (Days 5–6: PND 28–29) the animals received a daily portion of water in 2 pipettes, each containing 10 mL, within their home cage. This manner of water administration has been established as effective (Akirav et al., 2006; Belelovsky et al., 2007; Merhav and Rosenblum, 2008; Slouzkey et al., 2013; Awad et al., 2015). On Day 7 (PND 30), the rats underwent a retrieval session (T1) entailing 10-minute access to 2 pipettes of banana solution (each containing 10 mL), followed by 20-minute access to 2 pipettes of water (each containing 10 mL). This test served to examine the success of COA memory formation. To examine extinction of COA, we conducted 2 additional identical sessions (T2, T3) over the next 2 days (Day 8–9, PND 31–32).

For remote memory COA tests, animals received water ad libitum a few hours after the conditioning on Day 4 (PND 27). After 28 days (PND 55), surgery was performed, after which rats were deprived of water for 24 hours and then allowed 2 days of water baseline by 2 pipettes of 10 mL each as described earlier to habituate to water drinking from pipettes. This was followed by 3 days of tests (T1–T3, PND 58–60), performed as described for recent COA tests.

The aversion index, measured on the 3 testing days, was defined as millilters of water consumption/total fluid consumption (water + banana) × 100, with a maximal score of 100 for strong COA and a score of 50 indicating no COA.

#### CFC Acquisition and Extinction

**—**On Day 1 (PND 27), the previously operated rats, as described earlier, were placed in a conditioning chamber (Panlab, Spain) with a grid floor, black methacrylate walls, and transparent front door. For conditioning, they received three 0.35- to 0.4-mAMP, 0.5-second foot shocks delivered through the grids and administered 90, 210, and 330 seconds after the beginning of the session.

For recent CFC tests, the animals were placed in the context in which conditioning took place for 5 minutes on the day after conditioning (PND 28; T1) for a retrieval test and for 10 minutes on the following 2 days (PND 29–30; T2–T3) to induce extinction.

For remote CFC tests, a month after conditioning, the cannulated animals, as described earlier, were placed in the context where conditioning took place for a 5-minute retrieval test and for 10 minutes on the following 2 days for extinction (T2–T3).

The level of freezing (i.e., cessation of mobility except for breathing) was measured by means of a high-sensitivity weight-transducer system connected to the grid floor using an analog signal generated in response to animal movement and transmitted to the software module for analysis. Freezing was represented as a percentage of the time the animal was in a state of freezing during the test. After each behavior session, the shock grids and walls were cleaned with 70% ethanol and dried with a paper towel.

### Drugs and Administration

To affect the retrieval process, the anesthetic lidocaine (4%; 0.04 g/1 mL saline; vehicle: saline; Sigma-Aldrich, Rehovot, Israel) was microinfused into the IL 15 minutes prior to the retrieval of either recent or remote memory.

To affect the consolidation process, the protein synthesis inhibitor anisomycin (100 µg/1 µL; dissolved in 1 M HCl, diluted in saline and adjusted to pH 7.5 with NaOH; vehicle: saline with identical amounts of HCL and NaOH; Sigma-Aldrich) was microinfused immediately after the first retrieval test at either recent or remote time points. This procedure was in accordance with our previous report (Awad et al., 2015). Control rats were microinjected with the appropriate vehicle into the IL at the same time as the experimental groups.

For the microinfusion of the drugs, the stylus was removed from the guide cannula, and a 28-gauge (0.014 outer diameter/0.007 inner diameter) injection cannula that extended 1.0 mm beyond the tip of the guide cannula was inserted. The injection cannula was connected via PE20 tubing to a Hamilton micro-syringe driven by a CMA/100 microinfusion pump (Carnegie Medicin, Stockholm, Sweden). Microinfusion was performed bilaterally, delivering a 0.5-µL volume over 2 minutes. The injection cannula was left in position for an additional 1 minute before withdrawal to minimize dragging the injected liquid along the injection tract. Two rats from the COA recent experiment were excluded due to cannula blockage.

### Histology

**Figure 1.**
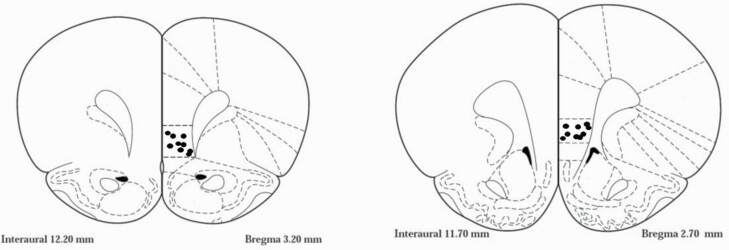
Schematic drawing illustrating cannulae placements representation of a sample of animals in the infralimbic cortex on coronal view at position +3.2 mm and +2.7 mm anterior to bregma (according to Paxinos and Watson, 1998). Black dots indicate the locations of the tips of the cannula.

Immediately after the completion of the last test, the animals were anesthetized, and 0.5 µL of Indian ink was microinfused into the IL-mPFC. Nissl staining was performed, and the cannula locations were examined under a light microscope. Figure 1 shows a schematic drawing of representative cannulae tip placement (black dots) in the IL-mPFC (coronal view at positions +3.2 mm and +2.7 mm anterior to bregma). An additional 2 animals were excluded from the analysis because the tip of 1 of their cannula was not in the IL-mPFC (as both should be in the right position).

### Collection of Tissue Samples and Western Blotting for c-Fos

Experimental animals were decapitated 90 minutes following recent (n = 8) or remote (n = 5) CFC retrieval. Unmanipulated home-cage animals were decapitated at the same age to serve as controls (n = 7 for each time point). Brains were quickly extracted, frozen in liquid nitrogen, and stored at −80°C until further analysis. The brains were sliced on a cryostat (Leica cm 1900) into 60-μm slices. Samples of the IL-mPFC were collected using a tissue cutter 1 mm in diameter (FST, Heidelberg, Germany) from slices at A/P = 3.2 mm from bregma. The obtained samples were homogenized in a lysis buffer (10 mM 4-(2-hydroxyethyl)-1-piperazineethanesulfonic acid (HEPES), 2 mM Ethylenediaminetetraacetic acid (EDTA), 2 mM Triethylene glycol diamine tetraacetic acid (EGTA), 1% phosphatase inhibitor, and 1% protease inhibitor; Sigma-Aldrich, Israel). Protein content was determined using the Bradford reagent assay (Bio-Rad). The samples were diluted in a Sodium dodecyl sulfate (SDS) sample buffer (1:1), boiled for three minutes at a temperature of 100°C, and stored at -80°C.

For western blotting, an equal amount of protein (10 µg) was loaded in each lane of sodium dodecyl sulfate–polyacrylamide gel electrophoresis (SDS-PAGE) gels. After standard electrophoresis, the proteins were transferred to a nitrocellulose membrane (0.45 μm; Bio Rad, Petah-Tikva, Israel) and the bands were visualized using Ponceau staining (Bio-Rad). Membranes were blocked for 1 hr at room temperature with a blocking buffer [3% blotting milk in Tris-buffered saline (TBS) containing 0.1% Tween 20 (TBS-T)]. Membranes were reacted with the primary antibody c-Fos rabbit mAb or actin (Cell Signaling Technology) overnight at 4°C. Following washes in TBS containing 0.1% Tween 20, the membranes were incubated with a corresponding secondary antibody (Cell Signaling Technology). Proteins were visualized by enhanced chemiluminescence (ECL Western Blotting Analysis System; GE Healthcare) and quantified with a charge-coupled device camera (XRS; Bio-Rad). Each sample was measured relative to the background, and total levels of c-Fos were normalized to levels of actin.

### Statistics

The outcome measures of aversion index for COA and percentage of time spent freezing for CFC are presented as mean and SEM. Two-way ANOVA with repeated measures was used to compare behavioral patterns between the groups (vehicle, anisomycin, and lidocaine) over the testing days, or 2-way ANOVA with groups and age of memory (recent vs remote) as factors followed by post-hoc test when appropriate. The data were analyzed using SPSS 21 software.

## RESULTS

Each of the 4 experiments (COA vs CFC, recent vs remote memory) included 4 groups of rats: lidocaine vs vehicle administered before T1 to target retrieval, and anisomycin vs vehicle administered after T1 to target consolidation. In each of the 4 experiments, no significant differences were found between the 2 vehicle groups, so they were merged into a single vehicle group for each experiment.

### IL-mPFC Does Not Control Retrieval or Extinction of Recent or Remote COA Memory

As expected, for both recent and remote COA, intake of banana solution on the conditioning day did not differ between the vehicle, anisomycin, and lidocaine groups (F_(2, 94)_ = 0.88, ns), as all groups had undergone the same manipulations up until that point.

**Figure 2.**
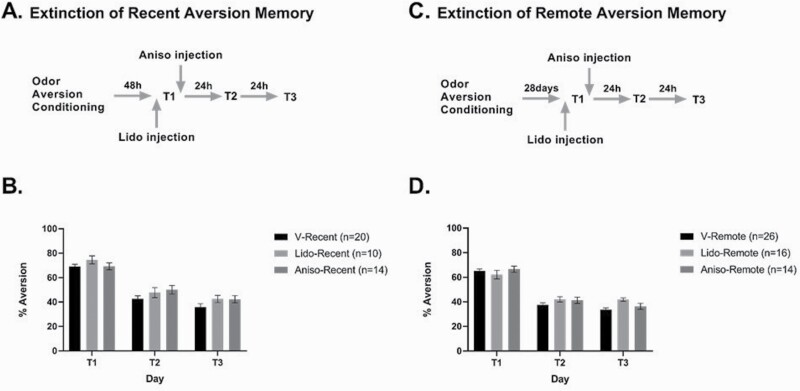
Inactivation of the infralimbic (IL) cortex by lidocaine or microinjection of protein synthesis inhibitor has no effect on retrieval or extinction consolidation of recent or remote odor aversion memory. (A, C) Schematic presentation of the experimental protocol for recent (A) and remote (C) COA memory and its extinction. (B, D) Animals’ IL cortex was infused with lidocaine (Lido) or vehicle 15 minutes before the first testing day (T1) to inhibit memory retrieval or with anisomycin (Aniso) or vehicle immediately after T1 to inhibit extinction consolidation. T1 occurred 2 days (recent memory, B) or 28 days (remote memory, D) after conditioning and was followed by 2 additional days of testing (T2, T3). On each testing day, animals were subjected to testing preference for the odorized water. Aversion index was measured as milliliters of water consumption/total fluid consumption (water + banana) × 100, with a maximal score of 100 for strong COA and a score of 50 indicating no COA. As aversion index did not differ between the 2 different vehicle groups, they were merged into a single group (V). Error bars show SEM. No significant differences were found between vehicle, lidocaine, and anisomycin groups.

For recent COA memory (Fig. 2B), 2-way ANOVA with repeated measures (drug × testing day) showed no significant effect of drug (vehicle [n = 20], lidocaine [n = 10], anisomycin [n = 14]; F_(2, 41)_ = 2.4, ns). Testing day (T1, T2, and T3) exhibited a significant effect (F_(1, 41)_ = 154, *P* < .001) but without any significant interaction with drug (F_(2, 41)_ = 0.64, ns), suggesting that all groups experienced similar extinction over the testing days.

For remote COA memory (Fig. 2D), 2-way ANOVA with repeated measures did not show differences between the vehicle (n = 26), anisomycin (n = 14), and lidocaine (n = 16) groups (F_(2, 53)_ = 1.66 ns). As for recent COA memory, testing day exhibited a significant effect (F_(1, 53)_ = 283, *P* < .0001), but without an interaction with drug (F_(2, 53)_ = 0.26, ns).

We also note that there was no difference in strength between recent and remote COA memory at T1 in the groups treated after T1 (vehicle and anisomycin [recent: 69.15 ± 1.53%, remote: 65.74 ± 1.3%; unpaired *t* test: t_(72) _= 1.69, ns]). Together, these results show that inactivation or blockade of protein synthesis in the IL-mPFC has no effect on retrieval or extinction of recent or remote COA memory.

### IL-mPFC Controls Extinction Consolidation, But Not Retrieval, of Recent CFC

During the pre-conditioning exploration phase of the recent experiment, an average freezing rate of 30.8% was observed consistent with random unconditioned movement. No difference between the vehicle, anisomycin, and lidocaine groups was observed in the exploration phase (F_2,38_ = 1.17, ns) or in the kinetics of conditioning, as tested by ANOVA for repeated measures (F_1,36_ = 1.31, ns) (see supplementary Fig. 1A).

**Figure 3.**
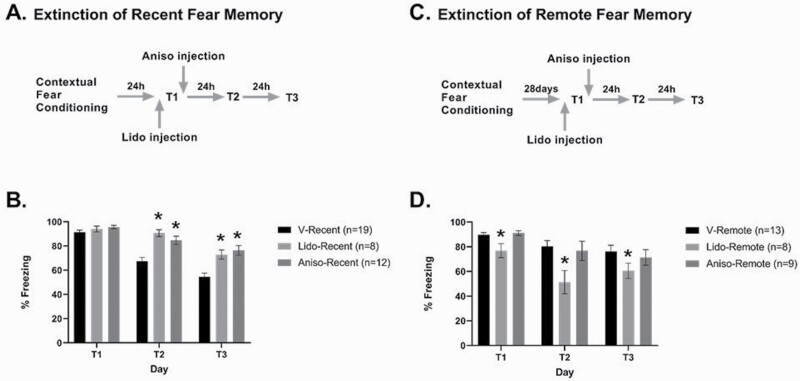
IL-mPFC controls extinction consolidation of recent fear memory and retrieval of remote fear memory. (A, C) Schematic presentation of the experimental protocol for recent (A) and remote (C) CFC memory and its extinction. (B, D) Behavioral results for animals whose infralimbic cortex was infused with vehicle (V), lidocaine (Lido), or anisomycin (Aniso) before or after T1, with infusion performed either 2 days (recent, C) or 28 days (remote, D) after conditioning. Infusion of lidocaine or anisomycin blocked extinction consolidation of recent CFC memory, and lidocaine infusion impaired retrieval of remote CFC memory. *Significantly different from the corresponding vehicle-treated group (*P* < .05).

For recent CFC memory (Fig. 3B), 2-way ANOVA for repeated measures on drug (vehicle [n = 19], lidocaine [n = 8], anisomycin [n = 12]) was conducted on all testing days (T1, T2, and T3), showing significant effects for drug (F_(2, 36) _= 14.8, *P *< .001), testing day (F_(1, 36) _= 161.5, *P *< .001), and interaction between drug and testing day (F_(2, 36) _= 4.0; *P *< .05). Post-hoc analysis showed that the 3 groups did not differ on T1 (ns), but the lidocaine and anisomycin groups exhibited significantly higher freezing levels compared with the vehicle group on T2 (*P* < .001) and T3 (*P* < .001); however, they did not differ from each other. These results indicate that inactivation of the IL-mPFC before T1 has no effect on recent CFC retrieval but that either inactivation or protein synthesis inhibition of the IL-mPFC impairs extinction of recent CFC memory (T2 and T3).

### IL-mPFC Controls Retrieval, But Not Extinction, of Remote CFC Memory

In the pre-conditioning exploration phase, an average freezing rate of 35.8% was observed, consistent with random unconditioned movement. No difference between the vehicle, anisomycin, and lidocaine groups was observed in the exploration phase (F_(2,29)_ = 0.34, ns) or in the kinetics of conditioning, as tested by ANOVA for repeated measures (F_(1,27)_ = 0.14, ns) (see supplementary Fig. 1B).

For remote CFC memory (Fig. 3D), 2-way ANOVA for repeated measures on drug (vehicle [n = 13], lidocaine [n = 8], anisomycin [n = 9]) on all testing days (T1, T2, and T3) indicated significant effects of drug (F_(2, 27)_ = 5.9, *P* < .05) and testing day (F_(1, 27)_ = 21.1, *P* < .001), but no significant interaction between drug and testing day (F_(2, 27)_ = 1.7, ns). Follow-up tests showed that the lidocaine-treated groups showed significantly less freezing than the groups treated with vehicle and with anisomycin (*P* < .05), while these 2 groups did not differ from each other. These results suggest that whereas activation of the IL-mPFC is critical for retrieval of remote CFC memory, protein synthesis in the IL-mPFC is not necessary for the consolidation of remote extinction memory. This suggests a role of IL-mPFC in the retrieval of the remote CFC memory but not in its extinction.

Finally, we note that there was no difference in the strength of recent and remote CFC memory at T1 (recent: 93 ± 1.3%, remote: 90 ± 1.1%; unpaired *t* test: t_(51)_ = 1.6, ns).

### Retrieval of Remote, But Not Recent, CFC-Induced IL-mPFC Activation

**Figure 4.**
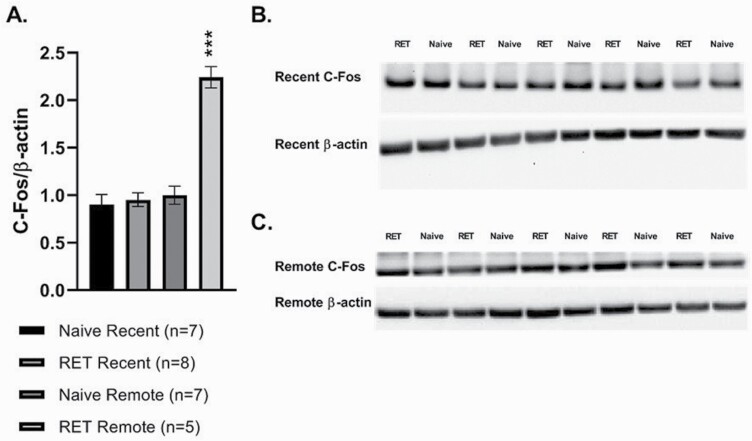
Retrieval of remote, but not recent, fear memory enhances IL-mPFC activation. (A) Two days (recent) or 28 days (remote) after animals aged PND 27 were fear conditioned, fear memory was retrieved by replacing the animals in the conditioning context. At 90 minutes after retrieval (RET), animals were killed, and expression of c-Fos protein in the IL cortex was quantified using western blotting. C-Fos levels were similarly evaluated in unmanipulated home-cage animals of the same ages. (B, C) Representative blots of c-Fos and actin expression within the IL of naïve and fear conditioned animals after recent (B) or remote (C) retrieval and their corresponding controls. ***Remote retrieval significantly enhanced c-Fos expression in the IL cortex compared with recent retrieval and naive (home-cage) groups (*P* < .001).

Because the results of inactivation by lidocaine suggest differential involvement of the IL-mPFC in recent and remote retrieval of CFC memory, in this experiment we aimed to determine if retrieval of recent and remote CFC memory induced different patterns of IL-mPFC activation, assessed through c-Fos expression (naïve recent [n = 7], recent CFC retrieval [n = 8], naïve remote [n = 7], remote CFC retrieval [n = 5]). Two-way ANOVA on c-Fos expression in IL-mPFC showed significant effects of group (naïve vs retrieval; F_(1, 23) _= 37.4; *P *< .001) and age of memory (recent vs remote; F_(1,23)_ = 43.2; *P *< .001) and a significant interaction between group and age of memory (F_(1, 23) _=_ _31.9; *P *< .001). Post-hoc analysis indicates that the remote retrieval group exhibited significantly higher c-Fos expression in the IL-mPFC than the other 3 groups (*P *< .001; Fig. 4).

## DISCUSSION

In this study, we first examined the ability of young animals to retain, retrieve, and extinguish recent and remote aversive memories while using 2 aversive paradigms based on aversion or fear memory. The second question we examined was whether recruitment of the IL-mPFC in these types of memory among post-weanlings is similar to our previous reports for adult animals (Awad et al., 2015). To examine this, we targeted the retrieval and extinction consolidation of COA and CFC by transiently inactivating the IL-mPFC with lidocaine before the first test (retrieval) or by inhibiting protein synthesis with anisomycin after the first test (extinction consolidation).

We found that post-weanling animals can form and retain COA and CFC memories for as long as 28 days after conditioning, as evidenced in their expression of robust memory. Moreover, post-weanlings did not show differences in the robustness of memory formed at recent compared with remote time points. Even though we did not directly compare between adults and post-weanlings, it seems that both recent and remote COA memories are weaker when learned at a young age (approximately 65% aversion; see Fig. 2) than as adults (approximately 95%; Awad et al., 2015), despite using the same procedure. This finding may be related to the crucial role of the basolateral amygdala for COA (Sevelinges et al., 2009a), which is only incorporated into the neural system that mediates odor-malaise association around weaning (Raineki et al., 2010) and is therefore not fully mature in juveniles. One interesting finding is that no such age difference was apparent for CFC, for which 80%–90% of freezing is present at T1 both in post-weanlings (Fig. 3) and in adults (Awad et al., 2015) tested at recent and remote time points. These data confirm recent reports (Shionoya et al., 2006; Raineki et al., 2012) indicating that CFC and inhibitory avoidance, another form of CFC (Travaglia et al., 2016), are acquired in rats as young as PND 24.

Nevertheless, until now, the retention and retrieval of remote memories and the brain areas involved in post-weanling-trained animals has not been determined. Previous studies showed that recent CFC memory in adult animals initially depends on the hippocampus, but over time cortical areas such as the IL-mPFC become essential for maintaining context representations (reviewed in Maren et al., 2013). Interestingly, we found here that the IL-mPFC controls retrieval of remote but not recent CFC memory in post-weanlings as well based on results of c-Fos expression and inactivation of the IL-mPFC during retrieval.

Contrastingly, the role of the IL-mPFC in COA retrieval differs between adults and post-weanlings. Specifically, whereas we previously showed that in adults the IL-mPFC is activated by remote but not recent COA memory, and IL-mPFC inactivation affects both recent and remote COA retrieval (Awad et al., 2015), we found here that in post-weanling animals, IL-mPFC inactivation has no impact on either recent or remote COA retrieval. These findings suggest that different networks mediate retrieval of COA memory in post-weanlings and in adults.

In adult animals, the IL-mPFC plays a pivotal role in extinction of fear memory through its connections with the amygdala as well as the hippocampus (Maren et al., 2013). Using a protein synthesis inhibitor to target the post-retrieval consolidation phase, we showed that the involvement of the IL-mPFC in extinction consolidation of recent CFC in post-weanlings is similar to what we and others reported for adult animals, resulting in impaired extinction (Table 1; Kim et al., 2009; Laurent and Westbrook, 2009; Sierra-Mercado et al., 2010; Kritman and Maroun, 2013; Awad et al., 2015). This finding further generalizes the role of the IL-mPFC in the consolidation of CFC extinction, as reported for extinction of cued conditioning (Santini et al., 2004; Kim et al., 2009). Yet unlike in adult animals, in juvenile animals the IL-mPFC is not critical for extinction of remote CFC memory (Table 1; Einarsson and Nader, 2012; Stern et al., 2014; Awad et al., 2015). These findings suggest that the storage of remote CFC extinction memories may rely on other cortical or subcortical regions. Moreover, the fact that IL-mPFC serves to inhibit recent CFC memory (through consolidation of extinction) but also to augment remote CFC memory (though expression) suggests in juveniles a time-dependent change in the role of IL-mPFC from inhibiting to augmenting fear, depending on the timing of retrieval.

**Table 1.** Comparisons between the effects obtained in adult (Awad et al., 2015) and juvenile animals (present study)^*a*^

Treatment		Juveniles (present results)		Adults (Awad et al., 2015)	
	Age at training	PND 27		~PND 60	
	Age at test	PND 28-30 (recent)	~PND 60 (remote)	~PND 60 (recent)	~PND 90 (remote)
Lidocaine in IL-mPFC before T1	COA retrieval	0	0	ä	ä
	CFC retrieval	0	æ	–	–
Anisomycin in IL-mPFC after T1	COA extinction consolidation	0	0	æ	0
	CFC extinction consolidation	æ	0	æ	æ

Abbreviations: 0, no effect; æ, impairment; ä, enhancement; –, not determined.

^*a*^Note that remote memory in post-weanlings-trained rats was evaluated at the same age as recent memory in adult-trained rats (approximately postnatal day 60).

We also show that the IL-mPFC does not participate in COA memory extinction in juvenile-trained animals, in contrast to its role in adult animals (Awad et al., 2015), again suggesting that different networks support COA memory in juveniles and in adults, as previously suggested for pre- and post-weanling animals (Raineki et al., 2009).

It is important to note that remote memory in juvenile-trained animals was assessed at the same age as recent memory in adult-trained rats (~PND 60; Table 1). We found that none of the IL-mPFC treatments that affected recent memory in adults—lidocaine for COA retrieval or anisomycin for CFC and COA extinction memory—had an effect on the remote memory of juvenile-trained animals (Table 1). This suggests that the age at training is a more important factor than the age at test to determine the role of the IL-mPFC in CFC or COA. This dissociation clearly shows that different systems are engaged in these memory processes and may suggest a shift from subcortical to cortical structures during the transition from juveniles to adults, corresponding to the late maturation of the frontal cortex during adolescence (Spear, 2000; Teicher et al., 2003).

The mPFC in general, and the IL-mPFC in particular, exhibits continuous structural and functional development throughout juvenility and up to adulthood (for review, see Delevich et al., 2018; Klune et al., 2021). Of particular relevance to the current work, the connections from the mPFC to the BLA begin to form between PND 10–15 and are still undergoing increases in fiber density and synaptic strength until PND 45 (Bouwmeester et al., 2002a, 2002b; Arruda-Carvalho et al., 2017).

In this study, we demonstrated that the IL-mPFC plays a differential role in retrieval and extinction of recent and remote CFC and COA among juveniles, in contrast to our previous findings for adults (Awad et al., 2015). These results resemble previous studies from our laboratory showing that memory is supported by different mechanisms in juvenile and adult animals (Schayek and Maroun, 2015; Slouzkey and Maroun, 2016).

## Supplementary Material

pyac012_suppl_Supplementary_Figure_S1

pyac012_suppl_Supplementary_Legends

## Interest Statement

None. The authors declare no financial or biomedical conflicts.
