# Supplementary figures and images for: Differential Recruitment of the Infralimbic Cortex in Recent and Remote Retrieval and Extinction of Aversive Memory in Post-Weanling Rats
Source: Int J Neuropsychopharmacol. 2022 Feb 4;25(6):489–97. doi: 10.1093/ijnp/pyac012 (PMC9211009; doi:10.1093/ijnp/pyac012)

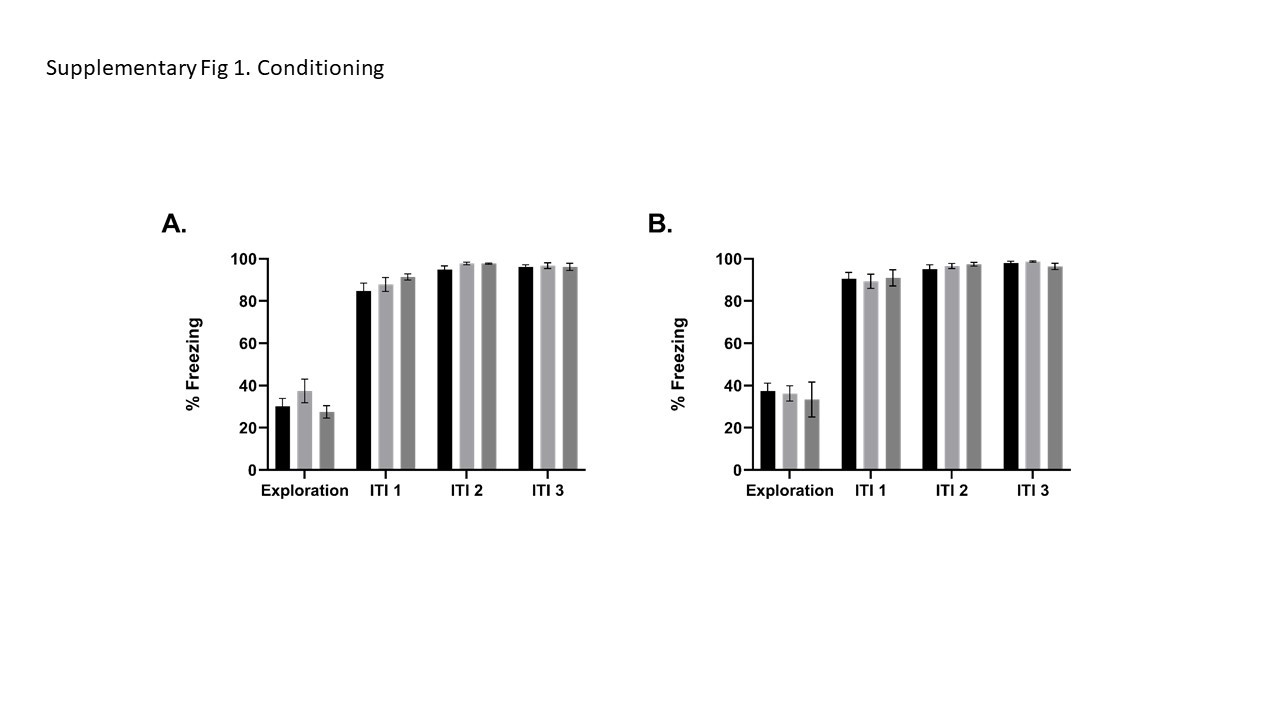

Supplement: pyac012_suppl_Supplementary_Figure_S1 [file pyac012_suppl_supplementary_figure_s1.jpeg]
