# Supplementary material for: Differential Recruitment of the Infralimbic Cortex in Recent and Remote Retrieval and Extinction of Aversive Memory in Post-Weanling Rats
Source: Int J Neuropsychopharmacol. 2022 Feb 4;25(6):489–97. doi: 10.1093/ijnp/pyac012 (PMC9211009; doi:10.1093/ijnp/pyac012)
Supplement: pyac012_suppl_Supplementary_Legends [file pyac012_suppl_supplementary_legends.docx]

**Supplementary Figure 1.** Freezing levels during pre-conditioning and shock-induced fear conditioning of juveniles rats that will be tested 2 days (A) or 28 days (B) later.
